# Detecting bracoviral orthologs distribution in five tsetse fly species and the housefly genomes

**DOI:** 10.1186/s13104-020-05161-8

**Published:** 2020-07-02

**Authors:** Kelvin M. Kimenyi, Muna F. Abry, Winnie Okeyo, Enock Matovu, Daniel Masiga, Benard W. Kulohoma

**Affiliations:** 1 Centre for Biotechnology and Bioinformatics, University of Nairobi, P.O. Box 30197, Nairobi, 00100 Kenya; 2 International Centre of Insect Physiology and Ecology, P.O. Box 30772, Nairobi, 00100 Kenya; 3 Makerere University, P.O. Box 7062, Kampala, Uganda

**Keywords:** Bracoviruses, Endogenous viruses, Eukaryotes, Tsetse fly, Housefly

## Abstract

**Objective:**

Mutualism between endogenous viruses and eukaryotes is still poorly understood. Several endogenous double-stranded polydnaviruses, bracoviruses, homologous to those present in parasitic braconid wasp genomes were detected in the tsetse fly (*Glossina morsitans morsitans*). This is peculiar since tsetse flies do not share a reproductive lifestyle similar to wasps, but deliver fully developed larvae that pupate within minutes of exiting their mothers. The objective of this study is to investigate genomic distribution of bracoviral sequences in five tsetse fly species and the housefly, and examine its value as a potential vector control strategy target point. We use comparative genomics to determine the presence, distribution across *Glossina* species genomes, and evolutionary relationships of bracoviruses of five tsetse fly species and the housefly.

**Results:**

We report on homologous bracoviruses in multiple *Dipteran* genomes. Phylogenetic reconstruction using within-species concatenated bracoviral orthologs shows great congruence with previously reconstructed insect species phylogenies. Our findings suggest that bracoviruses present in *Diptera* originate from a single integration event of the viral genome that occurred in an ancestor insect before the evolutionary radiation of different insect orders.

## Introduction

Mutualism between eukaryotes and viruses is rare, since most viruses have parasitic associations with their hosts [1–3]. A group of double-stranded DNA (dsDNA) viruses called polydnaviruses (PDVs) have symbiotic associations with thousands of parasitoid wasps (order *Hymenoptera*), which parasitize immunocompetent lepidopteran larvae to enable successful reproduction [4]. PDVs have co-evolved with wasps and present a unique opportunity to investigate genome rearrangements associated with these unique mutual symbiotic relationships [2, 5]. PDVs are broadly classified into two distinctly evolved genera: *Bracovirus* and *Ichnovirus* [6, 7]. Bracoviruses are common within a monophyletic group of wasps known as the Microgastroid complex [8]. It is thought that bracoviruses evolved from integration of a nudivirus into the genome of a Microgastroid complex ancestor approximately 100 million years ago (mya) [9]. Mutualism between wasps and bracoviruses developed over time, and functional association is estimated to date back to around 73.7 ± 10 mya [10]. Bracoviruses exist in two forms: a linear provirus integrated into the host genome that mediates vertical transmission as Mendelian traits, and as circular dsDNA virions [11, 12]. Viral replication, particle production and packaging into virions occur exclusively in a specialized part of the wasp ovaries (the calyx) [12], and precede injection alongside one or more wasp eggs into the parasitized caterpillar host during wasp oviposition [13]. Virions are replication deficient and their dsDNA is only expressed by the caterpillar host’s cellular replication machinery [6, 14]. The virion particles encode proteins that compromise the caterpillar host immune defense, thus preventing recognition, encapsulation and destruction of the parasitoid eggs and larvae [9, 15]. However, lack of genes that independently encode viral structural proteins has elicited a debate on whether bracoviruses are of viral origin or a ‘genetic secretion’ of the wasps [12]. An example is the bracoviral virion DNA in the wasp *Cotesia congregata* that consists of cellular genes of wasp origin, several viral genes and transposable elements [15]. Phylogenetic analysis of its functional bracoviral genes has highlighted sugar transporters of wasp origin [11]. Transfer of these wasp genes into the provirus was facilitated by transposable elements, and subsequently followed by co-evolution with the host’s genome, to become more specialized [2, 5, 9].

## Main text

The recently sequenced genome of the tsetse fly, *Glossina morsitans morsitans* (order *Diptera*), has revealed numerous homologous bracoviral genes (n = 305), widely spread across the genome; in addition to a large DNA hytrosavirus, the *Glossina pallidipes* salivary gland hypertrophy virus (GpSGHV) [16]. Although GpSGHV has been associated with reduced fecundity, life span, and causes salivary gland pathology in *Glossina*, its value as a potential entry point as a tsetse fly control strategy is has to-date not been explored. Perhaps more interesting is the finding of bracoviral sequences that bear close similarity (Basic Local Alignment Search Tool (BLAST), E values of < 1e−50) to those identified in the parasitic wasps (order *Hymenoptera*) *Glyptapanteles flavicoxis* and *Cotesia congregata*, where they occur as PDVs [2, 16]. Although the role of PDVs is well characterized in parasitic wasps, their organization, composition and functions in the tsetse fly genome is not known; indeed, their presence is new information. Molecular dating estimates that the orders *Diptera* (includes the tsetse fly and the house fly) and *Hymenoptera* (includes wasps) diverged ~ 350 mya [8], which is prior to the estimated date of first integration of bracoviruses into the ancestral wasp genome [9]. This raises the possibility that these genes may be remnants of PDVs acquired before this separation, and tsetse flies lost bracoviral mutualism after they adapted to larviparity (development of a single larva in its uterus as opposed to laying multiple eggs). An alternate hypothesis is that an undetermined braconid wasp may have parasitized the tsetse fly ancestor [16].

Tsetse flies are important vectors that transmit African trypanosomiasis to humans (sleeping sickness) and cattle (nagana). Approximately 70 million people and 50 million cattle are at risk of disease in tsetse-fly infested areas [17]. There are limited strategies for trypanosomiasis management primarily resulting from undesirable side effects of trypanocidal drug treatments; and there are emerging reports of multi-drug resistance [18–21]. According to the Pan African Tsetse and Trypanosomiasis Eradication Campaign (PATTEC), eradicating tsetse populations is the most viable approach of controlling trypanosomiasis in sub-Saharan Africa [22]. Identification of *Glossina* genes regulating vectorial capacity is thus a priority, as their manipulation would provide important clues for the development of effective vector control strategies, which will greatly facilitate trypanosomiasis control [18]. The tsetse fly, unlike other members of the order *Diptera*, does not lay eggs, but bears a fully developed larva (obligate adenotrophic viviparity) [23]. This makes it challenging to study PDVs in *Glossina* since during tsetse fly reproduction they are not replicated, excised from the host insect genome and packaged into viral particles that are mixed with semen like in parasitoid wasps. Wasp PDVs can easily be studied by first specifically extracting viral particles from the host [6]. Moreover, most bracoviruses consist of genes of host cell origin with protein domains conserved across metazoans, which further complicates analysis [24].

In this study, we aimed to identify polydnaviruses (PDVs) present in five recently sequenced tsetse fly genomes (*G. austeni, G. brevipalpis*, *G. fuscipes, G. m. morsitans,* and *G. pallidipes*) and the housefly (*Musca domestica*) [16, 25], by using references described in three parasitoid wasps (*Cotesia sesamiae Mombasa bracovirus, Cotesia congregata,* and *Glyptapanteles flavicoxis*) [26].

## Methods

### Identification of bracoviral orthologs

The proteomes of *G. austeni, G. brevipalpis*, *G. fuscipes, G. m. morsitans, G. pallidipes*, and *M. domestica* were retrieved from VectorBase (www.vectorbase.org) [27]. Bracoviral orthologs (n = 305) present in the *G. m. morsitans* proteome, previously described by the International Glossina Genome Initiative [16], were retrieved using a Perl script and used as sequence data references, in both nucleic and amino acid format (VectorBase assembly: GmorY1). The retrieved proteomes alongside previously described reference sequences were assigned to homologous clusters using OrthoMCL with default settings (BLASTP E-value cut-off 1e−5 and inflation index 2.5) [28]; using the *G. m. morsitans* proteome bracoviral orthologs (n = 305). Mapped orthologs were subsequently processed using BMX, as described in detail elsewhere [29–31].

### Sequence alignment and phylogeny reconstruction

Multiple sequence alignments were performed using MUSCLE [32]. Maximum likelihood (ML) phylogenetic analysis of the multiple aligned sequences with bootstrap values of 100 replicates was performed using PHYML version 3.520 [33]. Phylogenetic reconstruction using species-specific concatenated bracoviral orthologs was preceded by aligning sequences within individual ortholog cluster files to ensure joined sequences were of the same length.

## Results

**Fig. 1:**
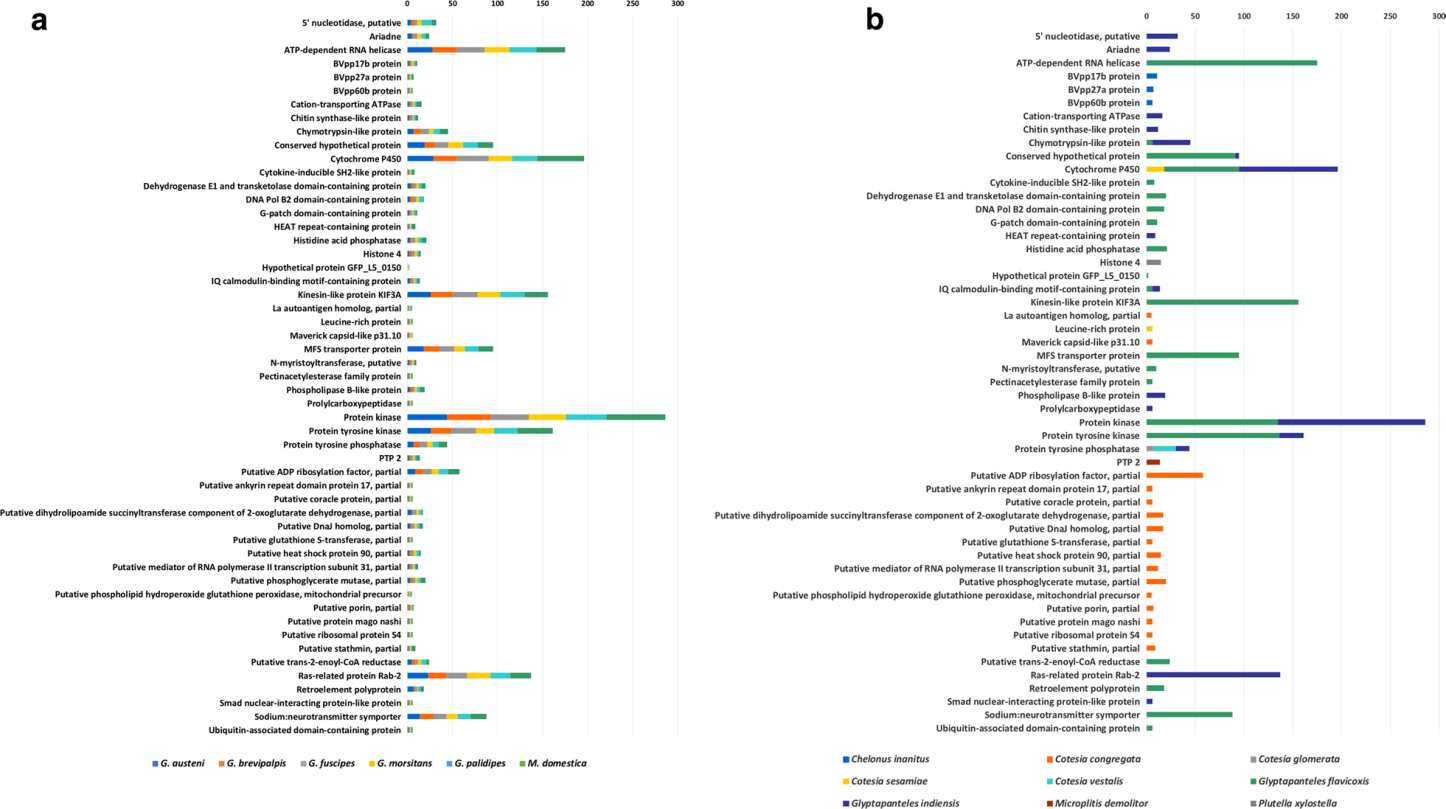
A total of 2020 bracoviral orthologs categorized into 53 clusters were identified in the five *Glossina* species and *M. domestica* genomes. **a** The distribution of orthologs identified across Dipteran insect species: *G. austeni* (n = 333)*, G. brevipalpis* (n = 303), *G. fuscipes* (n = 334), *G. morsitans* (n = 304), *G.* pallidipes (n = 332), and *M. domestica* (n = 414). **b** The distribution of orthologs by wasp species with homologous genes for each of the 53 clusters

**Fig. 2:**
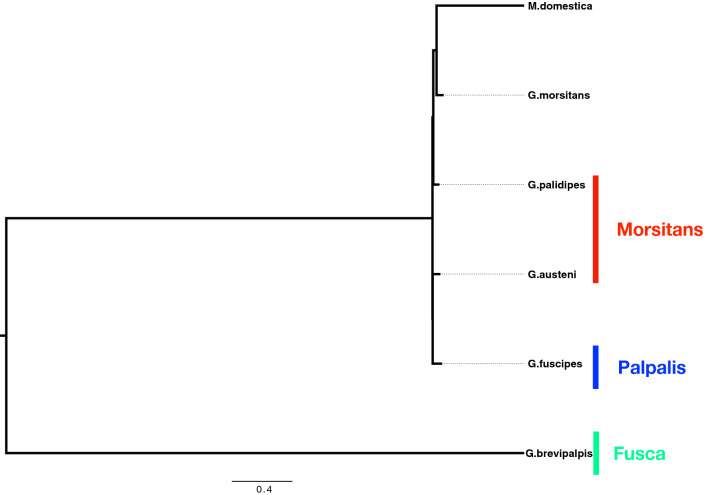
Phylogenetic reconstruction using species-specific concatenated bracoviral orthologs. There is congruence with previously reconstructed insect species phylogenies

We identified 53 bracoviral ortholog clusters, and a total of 2020 orthologs present across five *Glossina* species and *M. domestica* genomes (Fig. 1). The distribution varied across species: *G. austeni* (n = 333)*, G. brevipalpis* (n = 303), *G. fuscipes* (n = 334), *G. morsitans* (n = 304), *G.* pallidipes (n = 332), and *M. domestica* (n = 414). Most of the identified orthologs were homologous to those present in *Glyptapanteles indiensis* (n = 603) and *Glyptapanteles flavicoxis* (n = 1109). The protein kinase cluster had the most abundant number of orthologs (n = 286) (Fig. 1a), which were all homologous to those present in either *Glyptapanteles indiensis* or *Glyptapanteles flavicoxis* (Fig. 1b). We then established evolutionary relationships of bracoviral orthologs identified in the five *Glossina* species and *M. domestica* genomes by phylogenetic reconstruction using species-specific concatenated bracoviral orthologs. This revealed congruence with previously reconstructed insect species phylogenies [34]; tsetse fly species in the same group clustered closely together (Fig. 2). Interestingly, the concatenated bracoviral orthologs from *G. m. morsitans* appeared to be more distant to those from *G. brevipalpis* (same genus), compared to the distance to *M. domestica* (same order).

## Discussion

Bracovirus represent a unique symbiotic relationship between eukaryotes and endogenous viruses. Endogenous bracoviral sequences identified in the genomes of parasitoid wasps, some moth and butterfly lineages, and *Glossina morsitans morsitans* are plausibly due to rearrangements of ancient integrations. [12, 16, 35, 36].

We identified orthologs of polydnaviruses (PDVs) in recently sequenced *G. austeni, G. brevipalpis*, *G. fuscipes, G. pallidipes*, and *M. domestica* genomes. Although bracoviruses in wasps are co-opted to ensure their successful reproduction, their role in *Diptera* that do not share this mode of reproduction was unclear. Our findings suggest that PDVs are descended from a single ancestor after initial host integration before the evolutionary radiation of different insect orders, and their presence in the reference *G. m. morsitans* is not a single random genetic introgression event. It is unclear when exactly parasitoid wasp and *Dipteran* PDVs separated and diversified in their different hosts. Our findings support previous suggestions that bracoviruses are descended from a common ancestor in the Paleozoic Era, and raise the possibility of integration of PDVs before the separation of *Hymenoptera*, *Coleoptera*, *Lepidoptera* and *Diptera* [8]. We also show that PDVs vary in size and display phylogenetic diversity, which suggests intra-genomic PDVs rearrangements while co-evolving with the specific host’s genome to adapt to different environments. PDVs progressively decay after integration, with minimal effects on the host’s fitness, as they evolve blurring genetic detection [36, 37]. Fine-scale analyses of genetic variation underscore retention of adaptive alleles and loss of non-adaptive genes mediated through selection pressure at bracoviral genes [38]. Accumulation of mutations, recombination, and/or deletions leads to dissolution of bracoviral genes in the host genome, and genes that acquire function for the host are under positive selection [36]. PDVs that co-evolve with the recipient insect genome to provide new physiological function must adapt to the eukaryotic expression machinery [12, 35, 36]. Phylogeny reconstruction using concatenated within-species PDVs showed congruence with previously reconstructed insect species phylogenies [34], suggesting that adaptive bracoviral evolution within the order Diptera is associated with the host insect’s environment. It is intriguing to note that *G. m. morsitans* is more distant to *G. brevipalpis* than the housefly, which is of a different genus. *G. brevipalpis* is closest to the root, implying that its PDVs are the least diverse.

Understanding the genetic composition and organization of bracoviruses has led to new vector control strategies using transgenic approaches [12]. For example, the polydnavirus *Oryctes rhinoceros nudivirus* (OrNV) has been used as a biological control agent in palm tree farming against the rhinocerous beetle [12]. Successful delivery of bracoviral genes by wasps into lepidopteran larvae has also inspired notable agricultural applications. Currently, teratocyte secretory protein (TSP14) producing transgenic plants effectively reduce *Manduca sexta* growth and development, thus protecting the plants from insect damage [39]. Detrimental agricultural effects have also been observed, for example acquisition of horizontally transferred genes by lepidopterans, braconid wasps, and mites that detoxify inhibitory alkaloids and cyanide have increased pest fitness allowing to overcome plant defences produced upon attack [36, 40]. Our findings suggest that the presence of bracoviruses is neither a result of a pathogenic virus contamination of the reference *Glossina morsitans* genome, nor a single case of being parasitized by bracoviruses of wasp origin. This newfound knowledge provides better understanding of tsetse biology, and highlights possible novel intervention target points.

## Limitations

The limitation in this study was the absence of laboratory experimental validation using PCR of identified bracoviral sequences to those previously established in wasps. We anticipate that this would refine the number of orthologs to a smaller set of bracoviral homologs found in wasps. We were able to partly circumvent this challenge by using very stringent BLAST p-values (BLASTP E-value cut-off 1e−5 and inflation index 2.5).

## Data Availability

All materials and data used to perform this study are available in the main text. Raw sequence data used for analysis in publicly available at VectorBase (https://www.vectorbase.org/) with the following Gene set accession identities: *Musca domestica* (MdomA1.3), *Glossina morsitans* (GmorY1.9), *Glossina austeni* (GausT1.7), *Glossina brevipalpis* (GbreI1.7), *Glossina fuscipes* (GfusI1.7), and *Glossina pallidipes* (GpalI1.7). Orthologous reference sequences used (*Chelonus inanitus, Cotesia congregata, Cotesia congregata bracovirus, Cotesia glomerata bracovirus, Cotesia sesamiae Mombasa bracovirus, Cotesia vestalis bracovirus, Glyptapanteles flavicoxis, Glyptapanteles indiensis, Microplitis demolitor bracovirus, Plutella xylostella*) were first detected in *G. morsitans* and are available at PMID:24763584 or 10.1126/science.1249656.

## Abbreviations

DNA: Deoxyribonucleic acid
dsDNA: Double-stranded DNA
GpSGHV: *Glossina pallidipes* salivary gland hypertrophy virus
BLAST: Basic Local Alignment Search Tool
PCR: Polymerase chain reaction
PDVs: Polydnaviruses
OrNV: *Oryctes rhinoceros nudivirus*
TSP14: Teratocyte secretory protein
PATTEC: Pan African Tsetse and Trypanosomiasis Eradication Campaign

## References

[1] Burke GR, Walden KK, Whitfield JB, Robertson HM, Strand MR (2014). Widespread genome reorganization of an obligate virus mutualist. PLoS Genet.

[2] Espagne E, Dupuy C, Huguet E, Cattolico L, Provost B, Martins N, Poirie M, Periquet G, Drezen JM (2004). Genome sequence of a polydnavirus: insights into symbiotic virus evolution. Science (New York, NY).

[3] Federici BA, Bigot Y (2003). Origin and evolution of polydnaviruses by symbiogenesis of insect DNA viruses in endoparasitic wasps. J Insect Physiol.

[4] Strand MR, Burke GR (2012). Polydnaviruses as symbionts and gene delivery systems. PLoS Pathog.

[5] Jancek S, Bezier A, Gayral P, Paillusson C, Kaiser L, Dupas S, Le Ru BP, Barbe V, Periquet G, Drezen JM (2013). Adaptive selection on bracovirus genomes drives the specialization of Cotesia parasitoid wasps. PLoS ONE.

[6] Bezier A, Louis F, Jancek S, Periquet G, Theze J, Gyapay G, Musset K, Lesobre J, Lenoble P, Dupuy C (2013). Functional endogenous viral elements in the genome of the parasitoid wasp *Cotesia congregata*: insights into the evolutionary dynamics of bracoviruses. Philos Trans R Soc Lond B Biol Sci.

[7] Gundersen-Rindal DE, Pedroni MJ (2006). Characterization and transcriptional analysis of protein tyrosine phosphatase genes and an ankyrin repeat gene of the parasitoid *Glyptapanteles* indiensis polydnavirus in the parasitized host. J General Virol.

[8] Theze J, Bezier A, Periquet G, Drezen JM, Herniou EA (2011). Paleozoic origin of insect large dsDNA viruses. Proc Natl Acad Sci USA.

[9] Herniou EA, Huguet E, Theze J, Bezier A, Periquet G, Drezen JM (2013). When parasitic wasps hijacked viruses: genomic and functional evolution of polydnaviruses. Philos Trans R Soc Lond B Biol Sci.

[10] Whitfield JB (2002). Estimating the age of the polydnavirus/braconid wasp symbiosis. Proc Natl Acad Sci USA.

[11] Desjardins CA, Gundersen-Rindal DE, Hostetler JB, Tallon LJ, Fuester RW, Schatz MC, Pedroni MJ, Fadrosh DW, Haas BJ, Toms BS (2007). Structure and evolution of a proviral locus of *Glyptapanteles* indiensis bracovirus. BMC Microbiol.

[12] Bezier A, Annaheim M, Herbiniere J, Wetterwald C, Gyapay G, Bernard-Samain S, Wincker P, Roditi I, Heller M, Belghazi M (2009). Polydnaviruses of braconid wasps derive from an ancestral nudivirus. Science (New York, NY).

[13] Louis F, Bezier A, Periquet G, Ferras C, Drezen JM, Dupuy C (2013). The bracovirus genome of the parasitoid wasp *Cotesia congregata* is amplified within 13 replication units, including sequences not packaged in the particles. J Virol.

[14] Chevignon G, Theze J, Cambier S, Poulain J, Da Silva C, Bezier A, Musset K, Moreau SJ, Drezen JM, Huguet E (2014). Functional annotation of *Cotesia congregata* bracovirus: identification of viral genes expressed in parasitized host immune tissues. J Virol.

[15] Drezen JM, Bezier A, Lesobre J, Huguet E, Cattolico L, Periquet G, Dupuy C (2006). The few virus-like genes of *Cotesia congregata* bracovirus. Arch Insect Biochem Physiol.

[16] International Glossina Genome Initiative (2014). Genome sequence of the tsetse fly (Glossina morsitans): vector of African trypanosomiasis. Science (New York, NY).

[17] Simarro PP, Cecchi G, Franco JR, Paone M, Diarra A, Ruiz-Postigo JA, Fevre EM, Mattioli RC, Jannin JG (2012). Estimating and mapping the population at risk of sleeping sickness. PLoS Neglected Trop Dis.

[18] Barrett MP, Boykin DW, Brun R, Tidwell RR (2007). Human African trypanosomiasis: pharmacological re-engagement with a neglected disease. Br J Pharmacol.

[19] Brun R, Blum J, Chappuis F, Burri C (2010). Human African trypanosomiasis. Lancet.

[20] Wamwenje SAO, Wangwe II, Masila N, Mirieri CK, Wambua L, Kulohoma BW (2019). Community-led data collection using Open Data Kit for surveillance of animal African trypanosomiasis in Shimba hills, Kenya. BMC Res Notes.

[21] Wangwe I, Wamwenje SA, Mirieri C, Masila NM, Wambua L, Kulohoma BW (2019). Modelling appropriate use of trypanocides to restrict wide-spread multi-drug resistance during chemotherapy of animal African trypanosomiasis. Parasitology..

[22] Solano P, Ravel S, de Meeûs T (2010). How can tsetse population genetics contribute to African trypanosomiasis control?. Trends Parasitol.

[23] Benoit JB, Attardo GM, Baumann AA, Michalkova V, Aksoy S (2015). Adenotrophic viviparity in tsetse flies: potential for population control and as an insect model for lactation. Annu Rev Entomol.

[24] Bezier A, Herbiniere J, Serbielle C, Lesobre J, Wincker P, Huguet E, Drezen JM (2008). Bracovirus gene products are highly divergent from insect proteins. Arch Insect Biochem Physiol.

[25] Scott JG, Warren WC, Beukeboom LW, Bopp D, Clark AG, Giers SD, Hediger M, Jones AK, Kasai S, Leichter CA (2014). Genome of the house fly, *Musca domestica* L., a global vector of diseases with adaptations to a septic environment. Genome Biol.

[26] Dupuy C, Periquet G, Serbielle C, Bezier A, Louis F, Drezen JM (2011). Transfer of a chromosomal Maverick to endogenous bracovirus in a parasitoid wasp. Genetica.

[27] Giraldo-Calderon GI, Emrich SJ, MacCallum RM, Maslen G, Dialynas E, Topalis P, Ho N, Gesing S, VectorBase C, Madey G (2015). VectorBase: an updated bioinformatics resource for invertebrate vectors and other organisms related with human diseases. Nucleic Acids Res.

[28] Li L, Stoeckert CJ, Roos DS (2003). OrthoMCL: identification of ortholog groups for eukaryotic genomes. Genome Res.

[29] Abry MF, Kimenyi KM, Masiga D, Kulohoma BW (2017). Comparative genomics identifies male accessory gland proteins in five *Glossina* species. Wellcome Open Res.

[30] Abry MF, Kimenyi KM, Osowo FO, Odhiambo WO, Sewe SO, Kulohoma BW (2015). Genetic diversity of the Pneumococcal CbpA: implications for next-generation vaccine development. Human Vaccines Immunother.

[31] Kulohoma BW (2015). BMX: a tool for computing bacterial phyletic composition from orthologous maps. BMC Res Notes.

[32] Edgar RC (2004). MUSCLE: multiple sequence alignment with high accuracy and high throughput. Nucleic Acids Res.

[33] Guindon S, Gascuel O (2003). A simple, fast, and accurate algorithm to estimate large phylogenies by maximum likelihood. Syst Biol.

[34] Chen X, Li S, Aksoy S (1999). Concordant evolution of a symbiont with its host insect species: molecular phylogeny of genus Glossina and its bacteriome-associated endosymbiont, *Wigglesworthia glossinidia*. J Mol Evol.

[35] Desjardins CA, Gundersen-Rindal DE, Hostetler JB, Tallon LJ, Fadrosh DW, Fuester RW, Pedroni MJ, Haas BJ, Schatz MC, Jones KM (2008). Comparative genomics of mutualistic viruses of Glyptapanteles parasitic wasps. Genome Biol.

[36] Drezen JM, Josse T, Bezier A, Gauthier J, Huguet E, Herniou EA (2017). Impact of lateral transfers on the genomes of lepidoptera. Genes (Basel).

[37] Langley CH, Charlesworth B (1997). Endogenous proviruses as “mementos”?. Nature.

[38] Gauthier J, Gayral P, Le Ru BP, Jancek S, Dupas S, Kaiser L, Gyapay G, Herniou EA (2018). Genetic footprints of adaptive divergence in the bracovirus of *Cotesia sesamiae* identified by targeted resequencing. Mol Ecol.

[39] Maiti IB, Dey N, Pattanaik S, Dahlman DL, Rana RL, Webb BA (2003). Antibiosis-type insect resistance in transgenic plants expressing a teratocyte secretory protein (TSP14) gene from a hymenopteran endoparasite (*Microplitis croceipes*). Plant Biotechnol J.

[40] Tan CW, Peiffer M, Hoover K, Rosa C, Acevedo FE, Felton GW (2018). Symbiotic polydnavirus of a parasite manipulates caterpillar and plant immunity. Proc Natl Acad Sci USA.

